# Outcome and Predictors of Treatment Failure in Chronic Osteomyelitis Using Bioactive Glass Granules and Putty Formulations

**DOI:** 10.3390/antibiotics12121720

**Published:** 2023-12-13

**Authors:** Adriana Macedo Dell’Aquila, Gabriela Nagy Baldy dos Reis, Gabriel Trova Cuba, Walter Hamilton de Castro Targa, José Carlos Bongiovanni, Thomas Stravinskas Durigon, Mauro José Salles, Fernando Baldy dos Reis

**Affiliations:** 1 Infectious Diseases Discipline, Department of Medicine, Escola Paulista de Medicina (EPM), Universidade Federal de São Paulo (UNIFESP), São Paulo 04023-900, Brazil; ada.aquila@gmail.com (A.M.D.); gbtrova@gmail.com (G.T.C.); 2 Department of Orthopedics and Traumatology, Escola Paulista de Medicina (EPM), Universidade Federal de São Paulo (UNIFESP), São Paulo 04023-900, Brazil; gabriela.baldy@gmail.com (G.N.B.d.R.); thomas.durigon13@gmail.com (T.S.D.); fernando.baldy@unifesp.br (F.B.d.R.); 3 Institute of Orthopedics and Traumatology, Faculty of Medicine, Universidade de São Paulo, São Paulo 05403-010, Brazil; christarga2013@gmail.com; 4 Department of Orthopedics and Traumatology, Universidade de Mogi das Cruzes, Mogi das Cruzes 08780-911, Brazil; bongiova@uol.com.br

**Keywords:** chronic osteomyelitis, local treatment, bioceramics, bioactive glass, risk factors, recurrence, multidrug-resistant bacteria, Gram-negative bacteria

## Abstract

Background: The aim of this study is to evaluate the outcome of patients with cavitary chronic osteomyelitis undergoing adjuvant treatment with bioactive glass (BAG) S53P4 and identify the independent risk factors (RFs) for recurrence in 6- and 12-month patient follow-up. Methods: A retrospective, multicentre observational study conducted in tertiary specialised hospitals among patients undergoing the surgical treatment of chronic cavitary osteomyelitis using BAG-S53P4 in a granule and/or putty formulation to assess the clinical outcome and RFs for failure in 6- and 12-month patient follow-up. Results: Of the 92 and 78 patients with 6-month and 12-month follow-ups, infection was eradicated in 85.9% and 87.2%, respectively. In the 6-month follow-up, BAG-S53P4 in the granule formulation presented a greater risk of recurrence compared to the bioactive glass putty formulation or combined granules and putty (prevalence ratio (PR) = 3.04; confidence interval 95% [CI95%]: 1.13–10.52) and neoplasia (PR = 5.26; CI95%: 1.17–15.52). In the 12-month follow-up cohort of 78 patients, smoking (PR = 4.0; 95% CI: 1.03–15.52) and nonfermenting GNB infection (PR = 3.87; CI95%: 1.09–13.73) presented a greater risk of recurrence. Conclusions: BAG-S53P4 is a viable option for bone-void filling and the treatment of chronic cavitary osteomyelitis. Formulations of BAG with putty or in combination with granules showed better results.

## 1. Introduction

Postoperative osteomyelitis (POM) remains a fearsome complication following orthopaedic surgeries due to trauma. Osteomyelitis may rapidly evolve into a chronic process that is difficult to manage due to bacterial intracellular invasion into bone cells, progressing biofilm formation, persistent bony sequestrum, and ongoing bone resorption [1]. These particular features of chronic osteomyelitis (cOM) pathogenicity have been regarded as the likely reasons for local immune system evasion and higher rates of treatment failure using systemic antibiotic therapy [2].

Therefore, recent studies assessing the treatment of cOM with higher rates of success have highlighted the importance of surgical management, in which the nonviable bone is removed and the dead space within the affected tissue is filled with bone graft and/or bone substitute, which may inhibit local bacterial growth while also acting as a substrate for bone formation, thus preventing subsequent fractures [1]. It is important to consider a variety of factors when choosing a bone graft, including tissue viability, defect size, graft size, shape and volume, biomechanical properties, graft handling, cost, ethical considerations, biological characteristics, and associated complications [3]. Allografts, autografts, xenografts, and synthetic and biological materials are the main types of bone graft materials used. Each of these options comes with advantages and disadvantages that must be considered by the orthopaedic surgeon [4].

Indeed, the ideal bone substitute material should provide structural strength and enable bone recovery through osteoconduction, osteoinduction, and osteogenesis [5]. Osteogenesis exists when the graft material contains cells capable of synthesising new bone, so this property can only be achieved in autografts or when bone substitutes are enriched with cultured autologous cells. The gold standard for reconstructing small bone defects is autografts, which have excellent osteogenic properties [6]. Importantly, autografts have disadvantages, including pain and donor site morbidity, and other risks, such as major vessel or visceral injuries during harvesting [6]. Given the complications that allografts, xenografts, and autografts have, such as rejection, transmission of diseases, and cost, tissue engineering has been introduced in the last decade. A new scaffold and tissue graft can be designed using tissue engineering techniques to reduce the disadvantages of traditional grafts and enhance osteogenesis, osteoconductivity, and osteoinductivity [4].

Despite the traditional use of thermostable antibiotics incorporated into polymethylmethacrylate (PMMA) cement, its use is debatable due to the lack of osteointegration properties and the need for extra surgical procedures for removal and reconstruction surgery, leading to a risk of contamination at the surgical site [7]. Modern bioceramics and bone-void fillers with biodegradable properties to be used with or without antibiotics are currently available [1,8]. Bioactive glass (BAG) S53P4 (BonAlive^®^, Turku, Finland) represents a new generation of biomaterials that are able to induce osteoconduction and osteoinduction, producing a better biological interaction with bone tissue [4]. It consists of silicon dioxide (SiO_2_) (53%), sodium oxide (Na_2_O) (23%), calcium oxide (CaO) (20%) and pentoxide of phosphorus (P_2_O_5_) (4%) [9]. In addition to acting as a bone substitute, BAG-S53P4 has the ability to inhibit bacterial growth in vitro without the need for a local antibiotic drug [9,10], as it promotes an increase in local pH levels and a consequent increase in osmotic pressure caused by the exchange of alkaline ions with protons in the body fluid [10]. From the surface of its granule formulation, ions are released when in contact with biological fluids, thus affecting microbial growth in the surrounding environment. In vitro experiments have demonstrated an excellent antibacterial effect of BAG-S53P4 towards both planktonic and biofilm-forming bacteria, including those expressing multidrug-resistant profiles [8,10]. The selection between bioactive glass in putty or granules depends on the application and clinical requirements. Bioactive glass putty provides advantages, such as easy shaping for irregular bone defects, superior fit, and streamlined application during surgery. Its consistency enhances contact with bone tissue, potentially amplifying bioactive properties, and the uniform distribution contributes to consistent therapeutic effects. Improved adhesion and the potential for controlled release make putty formulations advantageous. The decision hinges on the surgeon’s preference, the characteristics of the bone defect, and the overall treatment strategy, with each formulation offering unique benefits for specific clinical scenarios and desired outcomes [11,12,13].

Despite all of the efforts in the surgical treatment of cOM with the use of local bone-void fillers with antibiotics, worryingly, rates of infection recurrence remain, including those associated with multidrug-resistant bacteria (MDR) in developing countries [14]. The aim of this study was to evaluate the clinical outcome of patients with cOM treated with BAG-S53P4 granules and putty formulations and to assess the predisposing risk factors (RFs) for treatment failure in 6- and 12-month patient follow-ups.

## 2. Materials and Methods

### 2.1. Study Design

This is a multicentre retrospective cohort study of a prospectively collected database of cases carried out over a two-year period (from April 2017 to November 2019) among two hospital centres specialised in the treatment of orthopaedic infections. The study included patients with a diagnosis of cOM who underwent surgical treatment with BAG-S53P4, with or without systemic antibiotic therapy, and was designed to assess the clinical outcomes and RFs for failure in a follow-up period of between 6 and 12 months. The surgical protocol for the treatment of cOM and the musculoskeletal infection team were the same for the two institutions, which applied similar empirical and antibiogram-guided antimicrobials and the same soft-tissue management and stabilisation techniques.

### 2.2. Participants/Study Subjects

All patients treated with BAG-S53P4 (BonAlive^®^, Turku, Finland) were identified through medical records by the service’s orthopaedics team. Patients of any age with a definitive diagnosis of cOM [15,16] were included in the protocol. Patients with a history of multiple soft-tissue surgical procedures in the site affected by osteomyelitis, with segmental bone lesions higher than 5 cm, and with concomitant septic arthritis were excluded. The study was approved on 19 February 2018 by the Research Ethics Committee of the hospital coordinating the study under protocol number 77277617.0.1001.5455.

### 2.3. Variables, Outcome Measures, and Data Sources

Data were collected by reviewing electronic medical charts. The clinical information consisted of demographic features, bones affected by the infection, patients’ comorbidities (diabetes, heart disease, neoplasia, paraplegia, tetraplegia, and thrombosis), antimicrobials relevant for prophylaxis, and empirical and antibiogram-guided therapies. In addition, information regarding the microbiological results of intraoperative sample collections and the duration of treatment and follow-up were collected. The patients were followed by the musculoskeletal infection team of each institution. The collected data were used to determine the primary outcome measure, which was the rate of recurrence of bone infection during follow-up.

### 2.4. Surgical Management

All patients underwent a comprehensive single-stage treatment, encompassing the removal of avascular bone and foreign materials, multiple intraoperative tissue samplings, the administration of culture-specific systemic antibiotics, stabilisation, dead-space filling, and primary skin closure.

Patients with implant-associated osteomyelitis underwent CT scans to check for healing. Those with complete bone healing underwent removal of the synthesis material, debridement of bone and adjacent tissue infected and devitalised, and filling of the dead bone space with BAG-S53P. Patients whose CT scans or surgery revealed the non-union of bone or bone fragility with a risk of fracture, after the removal of the synthesis material and bone and tissue debridement, underwent orthopaedic stabilisation using circular external fixation, filling the bone cavities with BAG-S53P.

We followed a well-established protocol to obtain multiple deep intraoperative samples. Sinus tracts were excised, and any infected implants were extracted. The infected region was meticulously exposed to facilitate the thorough resection of all necrotic bone. Excision proceeded until healthy, bleeding bone was revealed. Following the excision, the void within the affected tissue was filled with BAG-S53P4, with no additional material or antibiotics being introduced. Systemic antibiotic therapy was maintained for 6 to 12 weeks, guided by the final culture results.

### 2.5. Definitions

The diagnostic criteria for osteomyelitis included the following parameters: (1) “Acute osteomyelitis”, characterised by the detection of bone infection at the surgical site within 21 days after trauma, and “chronic osteomyelitis”, diagnosed beyond this period; (2) The outcome was categorised as “remission” when patients recovered without osteoarticular infection signs or symptoms, without the need for antibiotics or surgical intervention for bone infection. Patients experiencing treatment-related complications, such as new surgery, a second round of antimicrobial therapy, chronic antibiotic suppression, limb amputation, or persistent signs of osteomyelitis, were classified as having “treatment failure” [11]; (3) Polymicrobial bone infection was defined as the isolation of two or more microorganisms in at least one soft tissue or bone sample, while monomicrobial infection referred to the identification of a single pathogen in these cultures; and (4) Multidrug resistance (MDR) was characterised by acquired nonsusceptibility to at least one agent in three or more antimicrobial categories [14].

### 2.6. Specimen Collection and Microbiology

Following the thorough surgical debridement of the infectious focus, samples of soft tissue and/or bone were obtained, placed in appropriately labelled sterile vials, and subsequently dispatched to the hospital’s microbiology laboratory. There, traditional microbiological techniques were employed for culture and identification [14].

### 2.7. Statistical Analysis

For the statistical analysis, qualitative variables in the overall sample and groups categorised as failure and remission were described using frequencies and percentages. Quantitative variables were presented as medians and standard deviations (SDs). The relationship between variables was assessed through the Chi-square test, Fisher’s exact test, Poisson regression, Kaplan-Meier survival analysis, Cox regression model, and *t* test, as appropriate. Results with *p*-values below 0.05 (*p* < 0.05) were deemed statistically significant, ensuring at least a 95% confidence level in the conclusions presented.

## 3. Results

### 3.1. Study Population

**Figure 1:**
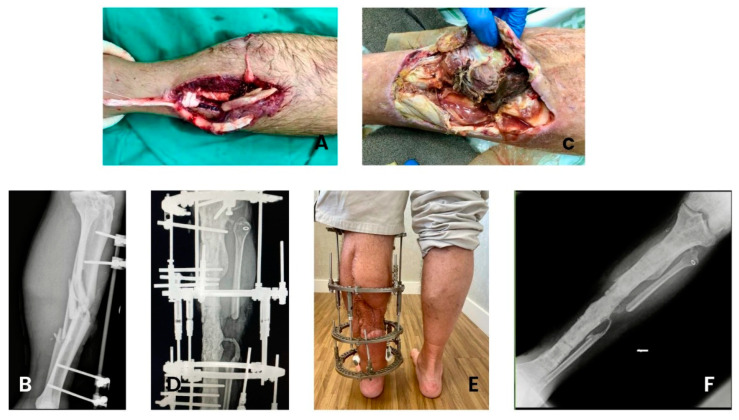
(**A**) Open Gustilo–Anderson IIIB fracture of the left tibia diaphysis. (**B**) Orthopaedic damage control with surgical cleaning, debridement, and linear external fixator. (**C**) Severe musculoskeletal infection with ischaemia and necrosis of fascia, muscles, and osteomyelitis. (**D**) Treatment of the bone defect with bioactive glass S53P4, bone lengthening, and linear external fixator. (**E**) Patient underwent soft tissue grafting and while remaining with circular external fixator. (**F**) X-ray showing osteointegration of bioactive glass S53P4 and removal of the external fixator.

During the study period, a total of 95 patients with cOM were included, but three patients were excluded from further analysis due to loss of follow-up. Therefore, the outcome analysis involved 92 patients, of whom 85.9% (79/92) and 87.2% (68/78) were in remission at the 6- and 12-month follow-ups, respectively. Briefly, 68.5% of the patients were male, with a mean age of 49.8 years (SD ± 21.1), and most were between 18 and 59 years of age. The main comorbidities were heart disease or hypertension (23.9%) and diabetes mellitus (21.7%). Implant-free osteomyelitis was diagnosed in 58.7% of patients. The presence of a fistula was diagnosed in 16.3% of patients, and the most affected bones were the tibia (22.8%) and femur (21.7%). Moreover, a clinical case of a patient with an open Gustilo–Anderson IIIB fracture of the left tibia diaphysis, which evolved into musculoskeletal infection (fasciitis and osteomyelitis) and significant bone loss, being treated with an external fixator, bone lengthening, and the insertion of active S53P4 bioactive glass, is shown in Figure 1. The demographic, clinical, and injury characteristics of the study population are summarised in Table 1.

### 3.2. Microbiological Findings

Regarding the microbiological findings, the culture was positive in 60.9% of patients, with Gram-positive bacteria yielded in 50.7%, mainly *Staphylococcus aureus* (27.5%), of which 26.3% was methicillin-resistant *Staphylococcus aureus* (MRSA). Among the Gram-negative bacilli (47.8%), *Pseudomonas aeruginosa* was the most frequently isolated (20.3%). Interestingly, 37.7% of the bacteria were MDR, mainly *Staphylococcus aureus* (38.5%). Polymicrobial infection occurred in 17.4% (12/69) of patients. The frequency of all microorganisms yielded from cultures is reported in Table 2.

Systemic antibiotic therapy was administered to all patients except for two. Among the prescribed antibiotics, teicoplanin, daptomycin, and meropenem were the most common, with 29.3%, 27.2%, and 22.8% of patients receiving them, respectively.

### 3.3. Six-Month Follow-Up Analyses (92 Patients)

In the univariate analysis, the nonfermenting GNB yield in the cultures was significantly associated with the recurrence of the infection (66.7% vs. 33.3%, *p* = 0.034) (Table 3). However, the presence of cancer (PR = 5.26; 95%CI: 1.17–23.73, *p* = 0.031) and the use of a bioglass granule formulation (PR = 3.44; 95%CI: 1.13–10.52, *p* = 0.030) were independent risk factors for recurrence in the multivariable analysis (Table 4). Patients treated with bioglass-based putty with or without granules in combination had a better outcome with lower recurrence rates (7.9%) when compared to the use of the granule formulation only (27.6%). The patient-, injury-, surgical-, and microbiological-related factors that were investigated for possible association with the treatment failure of cOM in the univariate analysis are shown in Table 3.

**Figure 2:**
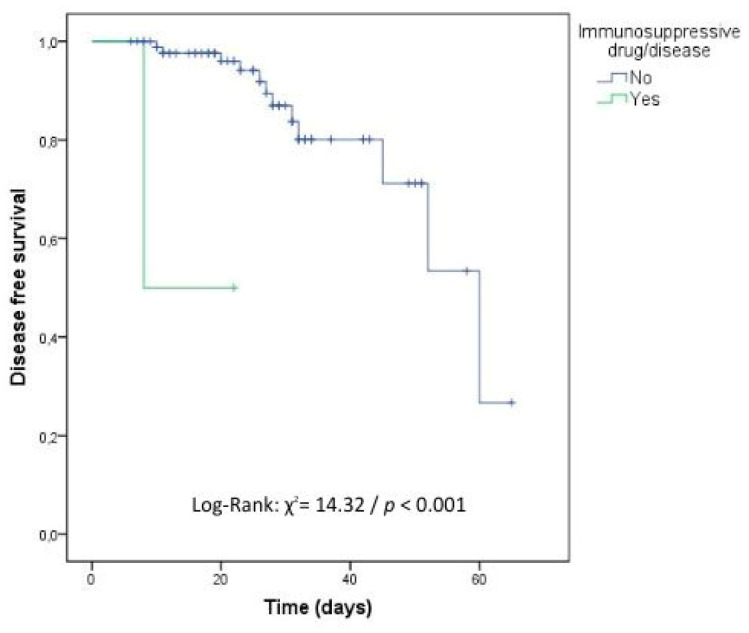
Kaplan–Meier survival curve for recurrence considering the presence of immunosuppressive disease and/or drug. DATABASE: 92 cases (No: 90 cases and Yes: 2 cases).

**Figure 3:**
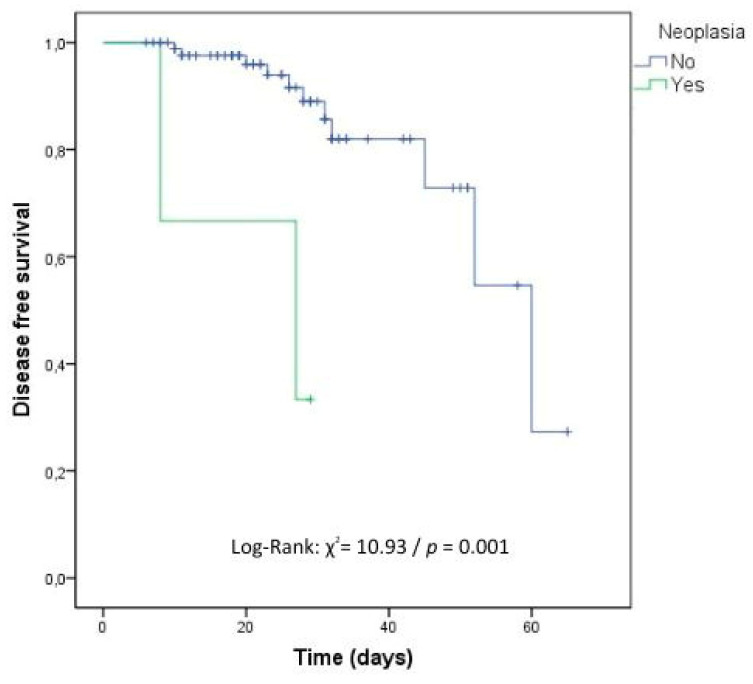
Kaplan–Meier survival curve for recurrence considering the presence of neoplasia. DATABASE: 92 cases (No: 89 cases and Yes: 3 cases).

**Figure 4:**
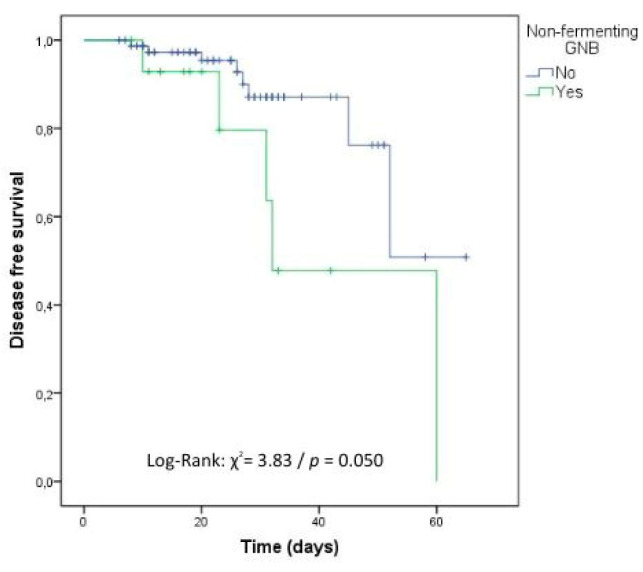
Kaplan–Meier survival curve for recurrence considering the presence of non-fermenting GNB. DATABASE: 92 cases (No: 77 cases and Yes: 15 cases).

In the survival analysis carried out to identify the independent risk factors related to time to recurrence of cOM, immunosuppressive drugs, the presence of cancer, and infection due to nonfermenting GNB negatively influenced the time to cOM with statistical significance (Table 4, Figure 2, Figure 3 and Figure 4).

### 3.4. Twelve-Month Follow-Up Analyses (78 Patients)

In the univariate analysis, nonfermenting Gram-negative bacilli agents isolated in the culture were significantly associated with the recurrence of the infection (33.3% vs. 9.1%, *p* = 0.042) and their presence remained an independent risk factor for the recurrence of cOM in the multivariable analysis (PR = 3.87; 95%CI: 1.09–13.73, *p* = 0.036) (Table 4). In addition, smoking habits were also an independent risk factor for recurrence (PR = 4.0; CI95%: 1.03–15.52, *p* = 0.045) (Table 4). Smokers were four times more likely to experience a recurrence of the infection than nonsmokers, while cOM with a positive culture for nonfermenting GNB was 3.87 times more likely to experience recurrence.

## 4. Discussion

The conventional treatment for cOM consists of the debridement of affected tissue and the addition of polymethylmethacrylate (PMMA) with local antibiotics, requiring at least two procedural steps—the first to allocate the PMMA with antibiotics and the second to remove the PMMA—since it is not a biodegradable material [8]. Therefore, this treatment requires a longer hospital stay and provides an increased risk of complications due to the necessity of multiple surgeries [10]. Other disadvantages of using PMMA are the risk of thermal necrosis resulting from cement curing, antibiotic resistance due to the low-level elution of antibiotics over time, and the lack of spontaneous bone healing without spacer removal [8,17].

Systemic and local antibiotic therapy with surgical debridement as a treatment for osteomyelitis, despite being associated with a satisfactory success rate, may fail in over 20% of cases [2,18,19]. Osteomyelitis treatment is complicated by various factors, including antimicrobial resistance, antibiotic tolerance due to metabolic changes and/or biofilm formation, the difficulty of antibiotics to reach infected and damaged bone, the colonisation of potentially antibiotic-protected reservoirs within the bone’s sub-structure, and the inability of systemic antibiotics to concentrate at higher levels due to vascular damage, while cause systemic toxicity and side effects [2,18,19].

In vitro, BAG S53P4 has demonstrated antimicrobial activity against Gram-positive and Gram-negative bacteria, including MDR, and does not appear to select the resistance of microbial strains. Usually, the treatment of cOM with BAG requires only one surgical procedure. Therefore, the risk for complications is lower, the hospital stay is shorter, and healthcare costs are likely reduced [20,21]. In this case series, no patient underwent extra surgeries. However, it is also important to assess the RFs associated with the recurrence of infection among patients to better target the indications for treatment with bioglass and increase the success rate with the product.

According to current guidelines for the management of cOM, the ideal treatment requires surgical debridement, the use of local bioceramics with antimicrobial activity, and the administration of systemic antibiotics for a minimum of 6 weeks [21,22,23]. In the present study, only two out of ninety-two patients were treated with systemic antibiotic therapy, with teicoplanin and meropenem being the most prescribed drugs. Nevertheless, the treatment of cOM in one-stage surgery with the use of BAG-S53P4 eradicated 87.2% in the population with a 12-month follow-up. This eradication rate is similar to that found in most studies, including previous studies assessing larger population samples [8,24]. Interestingly, the bioglass-based putty showed a greater eradication of the infection when compared with the bioglass in granules. In addition, the putty presentation facilitates intraoperative handling, especially when filling the bone cavity, with less possibility of heterotopic calcification and fistulisation of the product [25].

Recent studies assessed the independent predisposing factors that may hamper bone healing and increase the risk of reinfection, including smoking habits, obesity, diabetes mellitus, malnutrition, and peripheral vascular disease [26]. Moreover, the contamination of surgical implants from the skin microbiota, poor conditions of the local skin, and soft tissues impairing adequate bone coverage; the prolonged duration of surgery including complex procedures for bone stabilisation due to open lower limb fractures using implants such as plates and screws or intramedullary nails; a lack of orthopaedic surgeon experience; inadequate postoperative wound care; the presence of hematoma at the surgical site; and the overall patient’s health and nutritional status or the presence of underlying medical conditions (diabetes, obesity, smoking habits) are factors playing a crucial role in determining susceptibility to postoperative osteomyelitis [14,26].

The impact of smoking on surgical outcomes is likely linked to the detrimental effects of recent inhalation, causing tissue hypoxia through vasoconstriction. This process is associated with insufficient stimulation of fibroblasts under conditions of oxidative stress. Consequently, there is a decrease in cell migration and an increase in cell adhesion, along with alterations to certain components of the cytoskeleton. This leads to the inadequate accumulation of connective tissue in the wound, resulting in delayed healing and an elevated risk of infection. A recent systematic review [27] demonstrated an increased risk of infection across 51 studies spanning various specialties. The meta-analysis revealed a significant rise in infection rates among the group of smokers. This study also found a statistically significant increase in relapse among the population followed for at least 12 months [28].

In in vitro studies, BAG-S53P4 proved to be effective for multiple agents in planktonic and biofilm bacteria [14,29,30]. On the other hand, a clinical study identified a higher risk of infection recurrence when polymicrobial infection due to *Pseudomonas aeruginosa* and *Staphylococcus aureus* was treated with Bioglass [24]. *Pseudomonas aeruginosa* is a Gram-negative bacterium that causes nosocomial infections as well as fatal infections in immunocompromised individuals. Recently, the World Health Organization recognised *P. aeruginosa* as one of the most life-threatening bacteria and listed it as a priority pathogen for new antibiotic development. Adaptability and intrinsic antibiotic resistance as well as *P. aeruginosa*’s ability to form biofilms limit the efficacy of systemic antibiotics. These highly structured biofilms are especially identified in patients with chronic infections, such as cOM, increasing morbimortality in these patients [31]. We observed that the isolation of nonfermenting Gram-negative bacilli in the culture, especially *Pseudomonas aeruginosa*, resulted in a higher risk of relapse in patients with a 12-month follow-up, regardless of whether it was associated with other microorganisms. It is not known whether the use of local antibiotics may contribute to the emergence of resistant strains and whether the presence of *P. aeruginosa* may predispose patients to a greater likelihood of relapse, regardless of the product used. None of the patients in this study needed local soft-tissue coverage, which represents one of the situations in which recurrence cases increase [22]. However, patients with neoplasia were included, showing a higher probability of recurrence at the 6-month follow-up.

The present study had several limitations. Firstly, it adopted a retrospective case-series design, conducted at specialised centres providing distinctive orthopaedic care to a regional population situated in a major city within a developing country. Consequently, the findings of this study might not be generalisable to other medical facilities. The cohort comprised diverse patients treated by various orthopaedic trauma surgeons, albeit with support from the musculoskeletal infection team for the entire group. Moreover, microbiological diagnosis and susceptibility tests were performed using different methodologies, while no molecular and genotypic analyses were performed to identify clonal variants or similar patterns of resistance mechanisms. However, this study identified many MDR-GNB infections, such as *Pseudomonas aeruginosa*, with a high frequency of XDR strains.

## 5. Conclusions

BAG-S53P4 is a bone substitute option for the treatment of chronic osteomyelitis, with high rates of infection eradication, especially for the putty with or without granules. However, when the presence of *Pseudomonas aeruginosa* is identified in the bone infection, the product should be used with caution, and the possibility of using combined systemic antibiotic therapy should be evaluated. Despite the need for a longer follow-up to assess its long-term beneficial effects, BAG-S53P4 is a good and well-tolerated bone substitute and can be used in the treatment of osteomyelitis with good primary results.

## Figures and Tables

**Table 1:** Demographic and clinical characteristics of the patients included in the study.

Demographics, Comorbidities, and Habits	N = 92 (%)
Gender	
Male	63 (68.5%)
Age (mean (range)) (Years old)	49 (34–64)
<17 years	5 (5.4%)
18–39 years	26 (28.3%)
40–59 years	35 (38.0%)
60–79 years	18 (19.6%)
>80 years	8 (8.7%)
Chronic heart disease and/or hypertension	22 (23.9%)
Diabetes mellitus	20 (21.7%)
Smoking habits	9 (9.8%)
Thrombosis	4 (4.3%)
Paraplegia or quadriplegia	4 (4.3%)
Neoplasia	3 (3.3%)
Immunosuppression condition	2 (2.2%)
Alcoholism	1 (1.1%)
Osteomyelitis	
Implant-free	54 (58.7%)
Implant-associated	38 (41.3%)
Pseudarthrosis	14 (15.2%)
Fistula	15 (16.3%)
Bioactive glass formulation	
Granules and putty	33 (35.9%)
Putty (only)	30 (32.6%)
Granules (only)	29 (31.5%)
Duration of infection	
<3 months	37 (40.2%)
>3 months	55 (59.8%)

**Table 2:** Patient characterisation according to the isolated agent in the culture.

Microorganisms	N = 69 (%)
Gram-positive cocci	35 (50.7)
* Staphylococcus aureus*	19 (27.5)
MRSA ^a^	5 (7.3)
MSSA ^b^	2 (2.9)
Coagulase-negative *Staphylococci* (CNS)	10 (14.5)
*Staphylococcus epidermidis*	4 (5.8)
*Staphylococcus caprae*	1 (1.4)
*Staphylococcus haemolyticus*	1 (1.4)
*Staphylococcus xylosus*	1 (1.4)
*Staphylococcus lugdunensis*	3 (4.3)
*Enterococcus avium*	1 (1.4)
*Enterococcus faecalis*	1 (1.4)
*Enterococcus faecium*	1 (1.4)
*Streptococcus acidominimus*	1 (1.4)
*Streptococcus viridans*	1 (1.4)
Gram-negative bacilli	33 (47.8)
*Pseudomonas aeruginosa*	14 (20.3)
*Escherichia coli*	5 (7.2)
*Klebsiella pneumoniae*	6 (8.7)
*Acinetobacter baumannii*	2 (2.9)
*Proteus mirabilis*	2 (2.9)
*Klebsiella oxytoca*	1 (1.4)
*Morganella morganii*	1 (1.4)
*Enterobacter aerogenes*	1 (1.4)
*Enterobacter cloacae*	1 (1.4)
*Candida albicans*	1 (1.4)
MDR ^c^	26 (37.7)
*Staphylococcus aureus*	10 (14.5)
*Pseudomonas aeruginosa*	5 (7.3)
*Klebsiella pneumoniae*	4 (5.8)
*Escherichia coli*	2 (2.9)
*Staphylococcus caprae*	1 (1.4)
*Staphylococcus epidermidis*	3 (4.3)
*Staphylococcus xylosus*	1 (1.4)

^a^ MRSA—methicillin-resistant *Staphylococcus aureus*; ^b^ MSSA—methicillin-sensitive *Staphylococcus aureus*; ^c^ MDR—multidrug-resistant bacteria: acquired nonsusceptibility to at least one agent in three or more antimicrobial categories.

**Table 3:** Univariate analysis of risk factors associated with treatment failure of osteomyelitis.

Characteristics	Remission No. (%) (N = 79)	Failure No. (%) (N = 13)	*p*-Value
Bioactive glass			
Putty (only)	28 (93.3%)	2 (6.7%)	0.058 *
Granules and putty	30 (90.9%)	3 (9.1%)	
Granules (only)	21 (72.4%)	8 (27.6%)	
Duration of infection			
<3 months	33 (89.2%)	4 (10.8%)	0.453 **
>3 months	46 (83.6%)	9 (16.4%)	
Gram-positive cocci			
No	51 (85.0%)	9 (15.0%)	1.000 *
Yes	28 (87.5%)	4 (12.5%)	
*Staphylococcus* spp.			
No	55 (85.9%)	9 (14,1%)	1.000 *
Yes	24 (85.7%)	4 (14.3%)	
Gram-negative bacilli			
No	57 (89.1%)	7 (10.9%)	0.204 *
Yes	22 (78.6%)	6 (21.4%)	
Nonfermenting Gram-negative bacilli			
No	69 (89.6%)	8 (10.4%)	0.034 *
Yes	10 (66.7%)	5 (33.3%)	

Note: Patient characteristics were summarised as frequencies and percentages or median and compared using the Pearson Chi-Square test (*) or Fisher’s exact test (**). All tests were two sided, and *p*-values of <0.05 were considered statistically significant.

**Table 4:** Multivariate Cox proportional hazard model of risk factors associated with treatment failure of cOM *.

Variables	6 Months		12 Months	
	PR (CI 95%)	*p*-Value	PR (CI 95%)	*p*-Value
Smoking	2.37 (0.55; 10.24)	0.248	4.0 (1.03; 15.52)	0.045 **
Nonfermenting Gram-negative bacilli	3.92 (0.45; 33.91)	0.215	3.87 (1.09; 13.73)	0.036 **
Neoplasia	5.26 (1.17; 23.73)	0.031 **	2.64 (0.12; 57.66)	0.538
Bioglass in granules	3.44 (1.13; 10.52)	0.030 **	3.04 (0.46; 19.84)	0.246

* cOM: chronic osteomyelitis; PR: prevalence ratio. ** Significance probabilities in multivariate analysis refer to Poisson regression.

## Data Availability

The data presented in this study are available on request from the corresponding author.
